# Bulbar urethral stricture: penile skin flap may be a good option?

**DOI:** 10.1590/S1677-5538.IBJU.2019.05.01

**Published:** 2019-01-29

**Authors:** Luciano A. Favorito

**Affiliations:** Unidade de Pesquisa Urogenital Universidade do Estado de Rio de Janeiro Hospital da Lagoa Federal Rio de Janeiro Brasil Professor Associado da Unidade de Pesquisa Urogenital da Universidade do Estado de Rio de Janeiro Urologista do Hospital da Lagoa Federal, Rio de Janeiro Editor Associado da International Braz J UrolBrasil

The September-October 2019 issue of the International Brazilian Journal of Urology presents original contributions with a lot of interesting papers in different fields: Infertility, Bladder Cancer, Prostate Cancer, Renal Cell Carcinoma, Partial nephrectomy, Kidney stones, Nocturnal Enuresis, Basic Research, Urinary Incontinence, Transplantation, UPJ Obstruction, Pelvic Organ Prolapse, Hypogonadism, Vasectomy, Herbal Medicine in Fertility and Urethral Strictures. The papers come from many different countries such as Italy, Brazil, USA, Turkey, China, France, Iran, Lebanon, Singapore, Colombia, Tunisia, India and Spain, and as usual the editor ´s comment highlights some papers. We decided to comment the paper about a very interesting topic: Penile skin flap for anterior urethral sctricture (1).

Doctor Hmida and collegues from the Sahloul Hospital Sousse, Tunisia, performed on page 1057 an interesting study about the Penile skin flap for anterior urethral stricture. They studied 77 patients underwent substitution urethroplasty using dorsal penile skin flap for bulbar urethral strictures. The mean stricture length was 5cm (3-10 cm) and the mean flap length was 6cm. The mean follow-up was 60 months (6-120). The overall success rate was 88%. The authors concluded that urethroplasty using penile skin flap appear to be a safe and efficient technique for the treatment of a long and complex anterior urethral stricture.

There are several options for the treatment of anterior urethral stricture (2-4). The patient’s stricture position, length and complexity are important factors to decide the surgical technique (5-7). For long bulbar strictures the buccal mucosa graft (BMG) as gold-standard material due to its histological characteristics and very good long term results (8-12). However, there are multiple situations whereby BMG is inadequate (prior buccal harvest) or inappropriate for utilization (heavy oral radiation). The fascio-cutaneous flaps could be a good option in these situations. The penile skin flap is easy to perform, do not need urethral mobilization and the present paper shows a success rate of 88%, a very significant number. We congratulate the authors for this very important contribution.
